# KidzMed e-learning to upskill student pharmacists to teach pill swallowing to children

**DOI:** 10.1371/journal.pone.0282070

**Published:** 2023-03-16

**Authors:** Alice P. McCloskey, Andrew Lunn, Michael J. Traynor, Emma J. Lim, Yincent Tse, Philippa G. McCabe, Ravi D. Mistry, Nicola Vasey, Ailsa Pickering, Adam P. Rathbone

**Affiliations:** 1 School of Pharmacy and Biomolecular Sciences, Liverpool John Moores University, Liverpool, United Kingdom; 2 School of Pharmacy and Biomedical Sciences, University of Central Lancashire, Preston, United Kingdom; 3 The Great North Children’s Hospital, Newcastle-upon-Tyne, United Kingdom; 4 Institute of Health and Society Newcastle University, Newcastle-upon-Tyne, United Kingdom; 5 Newcastle University Medical School, Newcastle-upon-Tyne, United Kingdom; 6 School of Computer Science and Mathematics Liverpool John Moores University, Liverpool, United Kingdom; 7 Newcastle University School of Pharmacy, Newcastle-upon-Tyne, United Kingdom; De Montfort University Faculty of Health and Life Sciences, UNITED KINGDOM

## Abstract

**Background:**

Appropriate medication use is essential in ensuring optimal pharmacotherapeutic outcomes. It is mistakenly assumed that adults can swallow solid oral dosage forms (SODFs, e.g. tablets/capsules colloquially referred to as ‘pills’), without difficulty and that children cannot. KidzMed is a ‘pill swallowing’ training programme designed to teach effective SODF use in patients of all ages. It may be utilised by healthcare professionals to assist patients taking SODFs. E-learning was essential for training during COVID pandemic to reduce viral transmission. The aim of this study was to explore UK student pharmacists views of e-learning to support swallowing solid oral dosage forms.

**Methods:**

This study used pre- and post-intervention online surveys on Microsoft Forms to evaluate self-directed eLearning about pill swallowing on MPharm programmes at three UK Universities using a 13-item survey. A combination of five-point Likert Scales and free-text items were used. The eLearning was available via the virtual learning environment at the University and embedded within existing curriculum. Descriptive statistical analysis was used to explore responses.

**Results:**

In total, 113 of 340 (33%) students completed the survey. Seventy-eight percent (n = 65) reported the eLearning would enable them to teach adults and children to swallow SODFs successfully. Learners either agreed or strongly agreed that they felt comfortable to teach patients (95%, n = 62/113) and parents or carers (94%, n = 60) to swallow medications having completed the e-learning. Student pharmacists generally found eLearning as an acceptable way to reflect on their own experiences of ‘pill’ swallowing and how to support patients to swallow SODFs.

**Conclusion:**

The KidzMed eLearning was well received by student pharmacists. Further work is needed to explore whether skills translates into real life application in the clinical settings.

## Introduction

In order to achieve effective medication, use and optimal therapeutic outcomes, a medicine should be acceptable and appropriate for the patient [1]. The oral route is considered patient friendly, with oral liquid medicines assumed most suitable, and commonly prescribed for children, and solid oral dosage forms (SODFs) or ‘pills’ for adults [2]. However, oral medications are not without their issues—liquids are typically expensive to manufacture, difficult to transport and store increasing the environmental impact of medications, and many are unpleasant tasting. There are also safety concerns with availability of different strengths leading to potential dosing errors, and use of certain excipients in children.

Regardless of medication formulations, healthcare professionals, including pharmacists, are now trained to offer appropriate formulations to meet an individual’s needs, and ‘make every contact count’ to optimise health outcomes [3]. Considerable time is spent counselling patients about ‘high-risk’ medicines or those requiring administration via a device, *e*.*g*., injections, inhalers and eyedrops. It is however incorrectly assumed for SODFs, that most patients can take these without difficulty unless they are particularly young, have dysphagia or a medical condition that leads to, or precipitates dysphagia. However, up to 40% of adult patients do not use SODFs as directed due to problems swallowing these formulations [4]. This situation has been defined as ‘pill aversion’–emphasising that rather than a physiological impairment to swallowing SODFs this is a cognitive phenomenon [5]. This may result in skipping doses or inappropriate modification to aid swallowing, and often goes unrecognised or unaddressed by healthcare professionals [6]. The European Medicines Agency suggests that swallowing SODFs is a learnt skill that can be taught to all patients from the age of three years [7]. Aversion to swallowing SODFs is a global issue affecting up to 54% of patients [8], and may be related to a lack of knowledge regarding effective swallowing techniques or previous negative experiences. Certain SODF physical characteristics can precipitate increased difficulty swallowing them, *e*.*g*., uncoated preparations, rough texture, large size (> 8 mm for tablets and size #0 (22 mm) for capsules) [2].

Nativ-Zeltzer and colleagues have developed and validated a short questionnaire (PILL5) that could easily be adopted for use in clinical settings as a quick diagnostic tool for ‘pill aversion’ [9]. This five-question screening tool enables the degree of pill dysphagia and localisation (if the ‘pill’ gets stuck in the chest or throat) to be determined. Patients reflect and self-report on their ‘pill’ swallowing experiences answering the questions on a five-point Likert scale with scores given to each option. The scores are used to identify those with ‘pill’ swallowing difficulties (those scoring >6/20), aiding their identification for interventions to improve medication use.

To date, children as young as three have been taught to swallow SODFs using the pop bottle method at the Great North Children’s Hospital Newcastle-upon-Tyne [10]. The multidisciplinary team have also developed KidzMed, an eLearning programme, and resources to train pill tutors and equip healthcare professionals with the necessary skills to teach ‘pill’ swallowing in their own clinical setting (accessed at https://www.e-Lfh.org.uk/programmes/kidzmed/). The e-learning takes approximately 20 minutes to complete, with additional time for simulated practice. Authors of this article (EL, YT, RM, NV, AP) were instrumental in the design and implementation of KidzMed. The programme reflects their paediatric healthcare backgrounds and wish for engaging training. Therefore, unlike other training materials, KidzMed is colourful and concise- designed to deliver key information in a user-friendly and time efficient manner. Adoption of colour, scrolling rather than clicking through traditional PowerPoint slides, and inclusion of short quizzes aids engagement for the duration of the learning time. Its successful implementation is staff and time efficient, requiring one member of the healthcare team spending approximately 15 minutes with a patient. The programme has been used in both face-to-face and virtual settings to teach student and qualified healthcare professionals [11]. It has also been used effectively to train children and young patient advisory groups directly [12]. The KidzMed programme is thus a useful tool for patients and student healthcare professionals alike, including pharmacists.

We had planned to include KidzMed in its entirety (eLearning plus live simulated practice) as part of our undergraduate pharmacy programmes, however, the onset of the coronavirus pandemic (COVID-19) meant that we could not implement this as intended. COVID-19 impacted education delivery globally, with the introduction of civilian lockdowns from March 2020 [13]. Universities struggled to deliver face to face teaching into 2021 and solely virtual learning became commonplace as an accepted delivery method for both traditional and novel curricula. In this article we report an educational intervention introducing the KidzMed eLearning resources to three UK universities (Liverpool John Moores (LJMU), Newcastle University (Newcastle) and University of Central Lancashire (UCLAN)) during the COVID-19 pandemic.

## Aim of the study

The aim of the study was to explore UK student pharmacists’ views of e-learning to support swallowing solid oral dosage forms.

The study posed the following research questions:

- What are student pharmacists’ exposure to content about pill swallowing in existing pharmacy curriculums?
- What are student pharmacists’ personal experiences of pill swallowing?
- What do student pharmacists think about e-learning to teach others how to swallow pills?

## Methods

The GREET checklist 2016 is used below to report the details (where applicable) of this educational intervention [14].

Ethical screening deemed the study minimal risk and approval was granted at institutional level for the three University sites involved (LJMU: PBS/2020-21/04, Newcastle: 403/2020, and UCLAN: HEALTH 0168).

### Intervention

Participation was entirely voluntary. The intervention used an online e-Learning for Health platform (eLfH) available through NHS Health Education England https://www.e-lfh.org.uk/programmes/kidzmed/. This included a single screen that learners scrolled down to identify areas of the curriculum, and pop-out and drop-down options for learners to interact with. Faculty did not provide any incentives to the students for completing the e-learning. However, the KidzMed e-learning does produce a certificate for any learner who completes it, which our students could include in their portfolio of evidence demonstrating continuing professional development (an essential assessment component across all three universities involved in the study).

We targeted students in their third year or above of the MPharm programme at the three study sites using a convenience sampling approach. All eligible students were provided with an electronic participant information sheet to read on their virtual learning page. This informed them about the study and what taking part involved. Student pharmacists were provided with instructions on how to complete the activities within a virtual learning page that they routinely accessed as part of the higher educational programme at their respective universities. The same page was shared between the three universities and embedded within existing module content. Students were provided with contacts to help with technical issues.

The Microsoft Forms platform was used to collect pre-learning and post-learning data. To participate, students had to select ‘Yes’ to a compulsory question on the pre-questionnaire stating that they had read the participant information sheet, were happy to complete the activities, and aware that once submitted, questionnaire data could not be retrieved as no personally identifiable information had been submitted. They could withdraw at any point during the study by closing their web browser and or failing to submit the questionnaires. Questionnaires were purposefully designed by the research team, piloted by six students -two from each study site and deemed fit for purpose. The pre-learning questionnaire included questions on learners’ demographics and their experiences of taking SODFs (using the validated PILL5 questionnaire of Nativ Zeltzer et al. which has proven criterion validity and reliability and is the first example of a SODF swallowing screening tool), as well as their previous educational experiences [9]. The post-learning questionnaire asked learners about the e-learning content in relation to length, complexity, favourite, and least favourite elements, as well as an opportunity to input free text comments about the resources.

Data was exported from Microsoft Forms into Microsoft Excel and a summary table generated using the COUNTIF and percentage functions. Quantitative analyses were performed on Microsoft Excel. Free-text comments were deductively, thematically analysed in Microsoft Excel using a framework approach to identify positive and negative comments about the eLearning by two authors (APM and APR). Word clouds were generated using RStudio from free-text comments. The packages tm [15] and SnowballC [16] were used to process the data by removing common stop-words and special characters. Following the processing steps, the package wordcloud [17] produced the illustrated word cloud with the size and colour of the words depicting the frequency and therefore importance of each word in the cloud.

## Results

**Fig 1:**
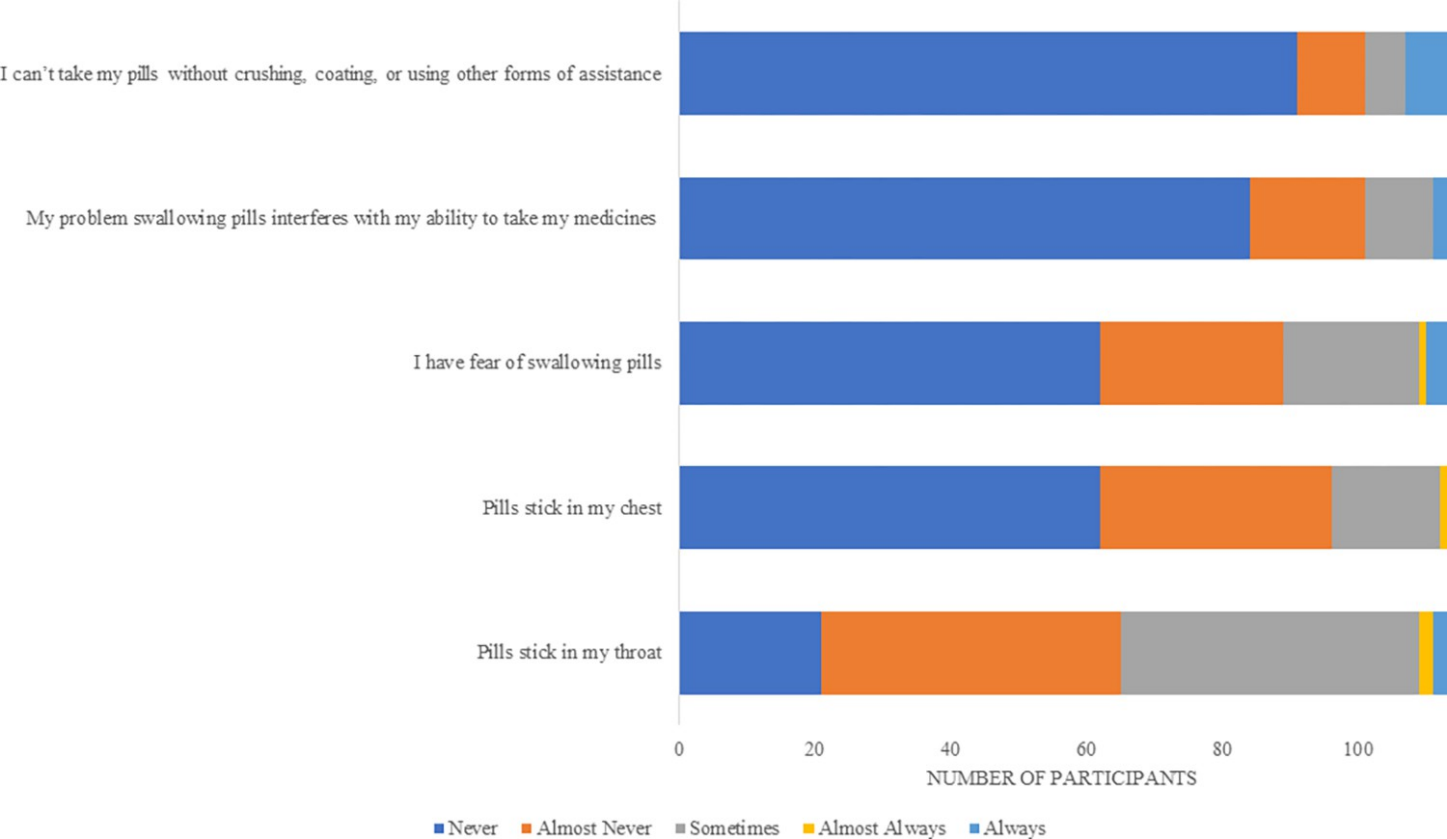
Learners’ personal experience of difficulties with SODFs (PILL5 Questionnaire).

A total of 113 responses out of a possible 340 students (33.2%) were returned for the pre-learning questionnaire and data is summarised in S1 Table. Learners identified themselves as women (77%, n = 87) and majority were in year 3 or 4 of pharmacy education (94%, n = 106). Fig 1 shows learners’ own experience using the PILL5 Questionnaire. Forty-two percent (n = 48) reported personal difficulties taking SODFs (*i*.*e*., “pills stick in my throat’ sometimes, almost always or always).

### Pre-intervention

**Fig 2:**
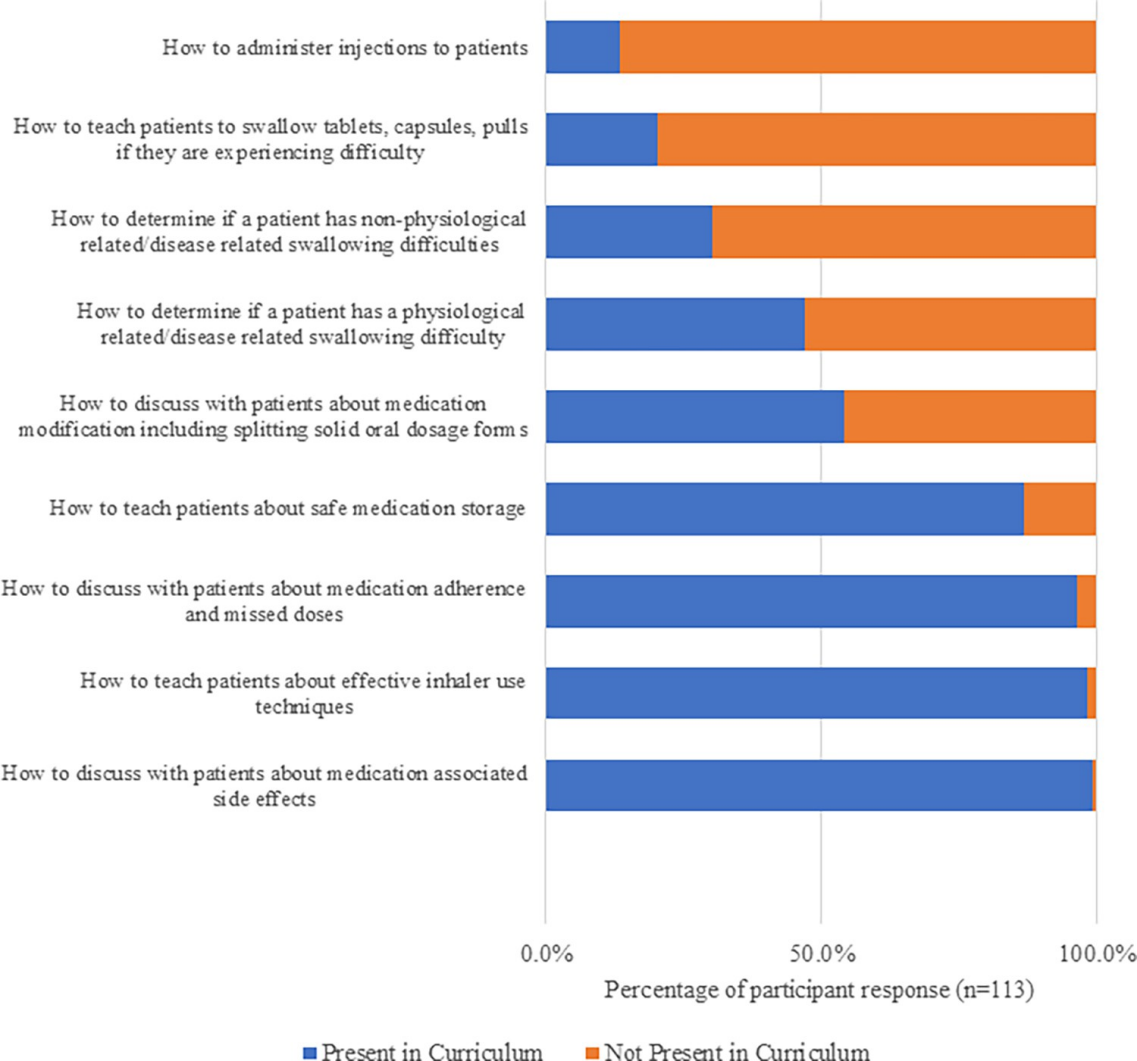
Prior learning in undergraduate pharmacy curriculum.

Learners’ prior exposure to teaching materials relating to pill swallowing is shown in Fig 2. Only 20% (n = 23) recalled learning about pill swallowing in existing curriculum.

### Post-intervention

Fifty-eight percent (n = 65) of learners returned post-intervention evaluation (S2 Table). Having completed the e-learning, 99% (n = 64/65) of respondents agreed or strongly agreed that teaching patients how to swallow SODFs was useful. All considered the e-learning has enabled them to teach others how to swallow SODFs—although 22% (n = 14/65) thought it was useful for teaching children only. Respondents agreed or strongly agreed they felt comfortable to teach patients (95%, n = 62) or parents or carers (94%, n = 60) to swallow SODFs. Eight-six percent (n = 55) agreed or strongly agreed that they would like to practice what they have learnt in a classroom environment before trying on patients.

**Fig 3:**
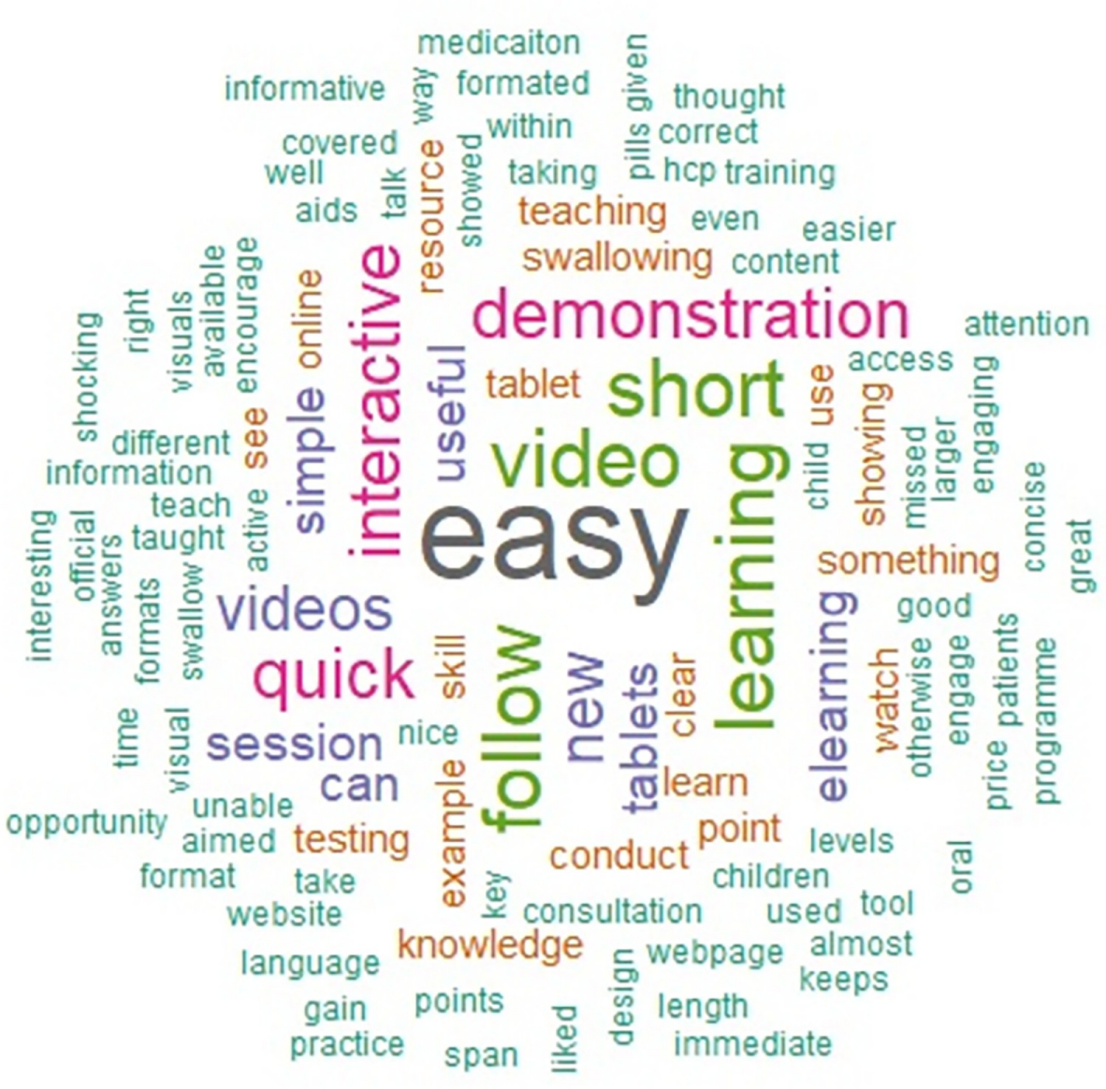
A Word Cloud generated using free-text responses about what learners liked most about KidzMed e-learning.

**Fig 4:**
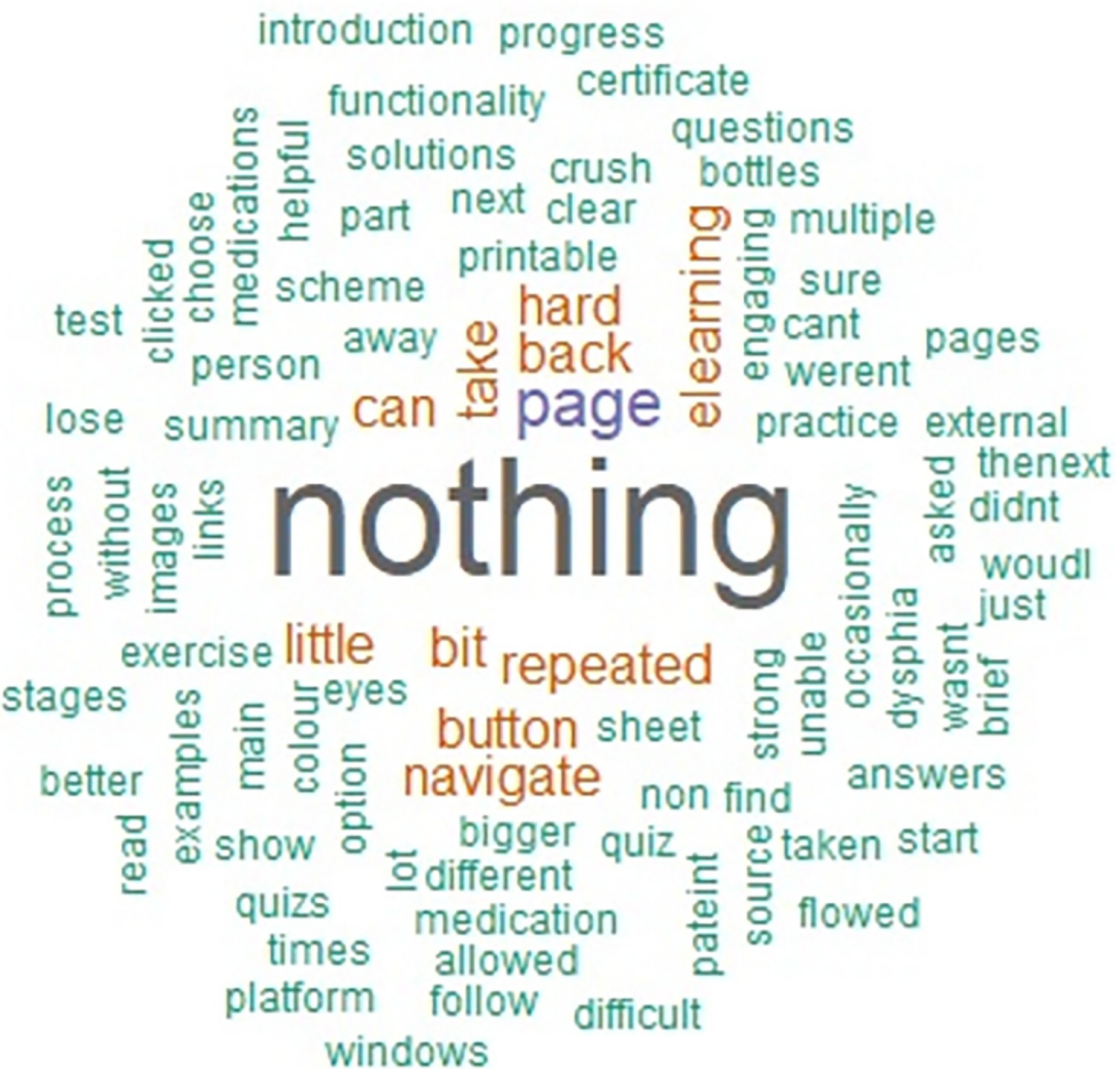
A Word Cloud generated using free-text responses about what learners liked least about KidzMed e-learning.

The free-text data from the questionnaire is summarised in the Figs 3 and 4 below.

Although a rudimentary form of analysis, the data suggested that students found the intervention easy to follow, quick and simple, particularly enjoying the interactive and video elements that demonstrated how to teach others to swallow SODFs.

The least preferred components were ‘the quiz’, which learners found challenging, and ‘the back button’ used to navigate the e-learning pages on the University platform, which occasionally prevented moving between pages. Additionally, student pharmacists indicated a preferences to supplement the e-learning with classroom practice before teaching real patients.

The complete data set is available in S3 Table.

## Discussion

Overall, this study indicates the KidzMed e-learning package to be an acceptable method to upskill student pharmacists how to teach adults and children to swallow SODFs during the COVID pandemic.

Self-reflection, as demonstrated in Fig 1, is a useful tool to determine the level of ‘pill aversion’ in student cohorts and also prompts students to put themselves in their patients’ shoes and recognise that if they have difficulties swallowing SODFs, then so too might their patients. The findings suggest a gap in UK pharmacy undergraduate curricula to support adults and children with swallowing SODFs (as per Fig 2). All pharmacy programmes across the UK must adhere to the regulatory body (The General Pharmaceutical Council’s) ‘Standards for the initial education and training of pharmacists’ [18]. Standards 5, 13, 26, 29, 37 and 49 refer to the safe and effective use of medicines (this captures all formulations, which largely include SODFs). Pharmacy is an increasingly patient-facing and clinical profession with the education and training standards reflecting this. Skills such as counselling on ‘pill swallowing’ and consideration of formulation choices at the point of prescribing and dispensing is important for future practice. According to student reports of previous learning, shown in Fig 2, it appears that existing curricula may wrongly overlook an important learning outcome, by assuming that SODFs do not require the same educational focus in terms of counselling as other routes of drug delivery or formulations delivered using devices.

Students liked the material and viewed it as relevant and a “useful skill to learn” for future practice (as per Fig 3). Students liked the e-learning’s user-friendliness, video demonstrations and webpage design. The resources may be generalisable and suitable for a broad range of learners and learning-styles [19]. A positive response to the quick nature of the e-learning also highlights that educational materials do not have to be lengthy in order to have impact. This e-learning tool has already been used successfully to teach healthcare students from other disciplines and qualified healthcare professionals [20]. Student pharmacists in this study also responded positively to the e-learning and expressed feelings of confidence to put their learning into practice with future patients, despite having little prior learning experience on this topic. Relevance of learning in real-life practice is important, in addition to application of learning in a safe environment. Students commented that in-person application would be worthwhile, echoing the literature and student desire for active in-person learning [21].

These findings are particularly relevant for educators of student pharmacists, and other healthcare professionals, as many of these students will work in patient-facing roles and encounter patients with pill aversion. The e-learning highlights the cost differences between liquid and SODF preparations alongside encouraging learners to reflect on the advantages and disadvantages of these oral formulations. Pharmacists are trained to integrate science with clinical practice which includes consideration of a medicine’s properties (size, texture, shape, storage requirements etc.) alongside the pharmacological action. Linking this knowledge with an awareness of pill aversion and drawing awareness to their own experiences of pill swallowing using a validated screening tool (*i*,*e*, PILL5), may prompt students in their future practice to enquire about SODF-taking abilities, identify those with difficulties, and inform next steps *i*.*e*., use the KidzMed method to train the patient, or consider alternative dosage forms.

A prime example of where this learning may be particularly relevant is for those working in infectious diseases. Patients with human immunodeficiency virus (HIV), for example, are required to take antiretroviral medications daily. The liquid preparations of these are notably unpalatable and the SODFs considered large and difficult to swallow, contributing to poor adherence [22,23]. Non-adherence to antiretroviral medications is problematic and can result in treatment resistant strains of HIV emerging, and associated morbidity and mortality for this patient cohort [24–26]. This argument could be extended more generally to adherence of antimicrobial medications, specifically liquid formulations where their storage, spillage and unpalatability may result in the medication course not being completed [27]. An ability to swallow SODFs also increases treatment options for those with conditions for which there are limited therapies available. Cystic fibrosis (CF) is an example where novel CFTR modulators such as Kaftrio® and Kalydeco® are only available as SODFs. Recently, at Alder Hey Children’s Hospital Liverpool ‘pill swallowing’ training was introduced to the CF clinic. CF patients aged 4–16 years were included and 10 patients successfully transitioned to SODFs with associated cost-savings and positive responses from children and carers [28]. An inability to swallow SODFs would prevent eligible CF patients from accessing these life-changing and life-saving medicines thus highlighting the necessity for this life-skill. Training healthcare professionals to support patients to overcome issues taking SODFs may therefore contribute to improved adherence, aid transition from liquid to SODFs, reduce the likelihood of emerging resistant strains and improve overall health outcomes. The findings thus provide evidence for the use of an e-learning package that when applied in the clinic, may have notable impact on patients’ lives, though further studies are needed to investigate this.

Beyond specific patient groups, supporting patients to swallow SODFs may also have significant cost and sustainability benefits—a bonus for financially stretched healthcare systems [29]. Although an economic evaluation has not been performed in these findings, learners’ confidence to support patients to swallow SDOFs may enable them to switch future patients from oral liquid or parenteral formulations to SODFs, an action that typically results in cost savings [10]. SODFs are typically more stable, easier to store and transport whilst also being more economically and environmentally favourable to produce than their oral liquid or parental counterparts. Further work is needed to evaluate the economic and environmental impact of the e-learning and whether it influences future practice of prescribing or supplying oral medication. However, evidence of such economic impacts is already emerging following application of KidzMed at The Great North Children’s Hospital Newcastle-upon-Tyne. Tse et al. have shown in their renal clinics that even within a short period of time (3 months) 25 patients could be switched to SODFs with proposed annual cost-savings of £46,588 [30]. Rashed et al. report similar findings following introduction of a ‘pill school’ to Evelina Children’s Hospital London [31]. Carers and patients were positive about the training and 24/30 (92%) of participants aged 3–14 years were discharged on SODFs with proposed cost-savings of over £31,000 per year. As outlined previously Alder Hey (Liverpool) also notice cost savings following introduction of KidzMed to the CF clinic [28]. The examples outlined provide evidence that teaching healthcare professionals and therefore patients to swallow SODFs has both patient and economic benefits, although the environmental implications of switching has yet to be explored.

This study has some limitations. Most learners identified as women, so this study may not have fully captured the experience of learners of other genders, however this reflects the gender balance of the pharmacy and healthcare workforce in the UK [32], but may not reflect the demographics of these groups globally. Also, evidence suggests that a higher proportion of females than males experience SODF swallowing difficulties [33]. This is attributed to females having smaller mouth cavities thus perhaps our findings over-estimate the extent of SODF swallowing difficulties in our student population. Our study took place during a global pandemic and our study population numbers are not considered high. This may be a result of lack of student engagement during this time. In addition, participation in the study was voluntary with no assigned grade or penalty for engagement or not, and the sample was taken from only 3/31 UK pharmacy schools. The findings and number of participants however reflect similar studies exploring ‘pill swallowing’ difficulties in adult patients/ student populations and thus are as likely to be as generalisable to the wider population [33].

Pooling together the views and learning experiences from three UK pharmacy schools indicates that perhaps there is a gap in learning and thus pharmacy educators nationwide can act on this. The overwhelmingly positive response from our sample of over 100 student pharmacists regarding the KidzMed e-learning is excellent feedback for the KidzMed indicates its utility for widespread integration in pharmacy curricula. There is an obvious drive for active learning among the sampled student population, and despite the timing of the study, students responded enthusiastically to a novel non-compulsory e-learning activity. Students can see the relevance of the KidzMed e-learning and completed the same training as qualified healthcare professionals who have applied this successfully in practice. This highlights that the learning is not simply a tick-box exercise, rather a skill that has real clinical application and benefits patients.

## Conclusion

This study shows that completing self-directed e-learning course on ‘pill swallowing’ allowed student pharmacists to reflect on their own experiences and learn how to support patients to successfully swallow SODFs. Students reported this was a useful skill to learn as part of their pharmacy training and expressed and interest to apply this in live sessions and in their future practice. This promising result should create future healthcare professionals who understand and can assist patients with difficulties, pill aversion, or those who do not know how to swallow SODFs thus broadening treatment options and improving medication use and therapeutic effects. Our work is the first example of using the KidzMed e-learning for student pharmacists in UK pharmacy schools. Further work is needed to evaluate students’ retention and application of knowledge and skills gained from the KidzMed e-learning package, and to explore its relevance and applicability to student pharmacists outside of the UK.

## Supporting information

S1 Table Summary of data from pre-learning questionnaire. (DOCX)

S2 Table Summary of data from post-learning questionnaire. (DOCX)

S3 Table Free-text responses from learners after completing the KidzMed e-learning. (DOCX)
