# Supplementary material for: KidzMed e-learning to upskill student pharmacists to teach pill swallowing to children
Source: PLoS One. 2023 Mar 16;18(3):e0282070. doi: 10.1371/journal.pone.0282070 (PMC10019696; doi:10.1371/journal.pone.0282070)
Supplement: S1 Table — (DOCX) [file pone.0282070.s001.docx]

# Supplementary Information Tables

| Table 1. Summary of data from pre-learning questionnaire |  | |  |
| --- | --- | --- | --- |
|  | n | % | |
| **Participants** | 113 | 100.0% | |
| Woman | 87 | 77.0% | |
| Man | 26 | 23.0% | |
| Non-binary | 0 | 0.0% | |
| Prefer not to say | 0 | 0.0% | |
| **Stage of study** | 113 | 100.0% | |
| First | 0 | 0.0% | |
| Second | 6 | 5.3% | |
| Third | 64 | 56.6% | |
| Fourth | 42 | 37.2% | |
| Fifth | 1 | 0.9% | |
| **Higher Education Institute** | 113 | 100.0% | |
| Newcastle University (Newcastle) | 46 | 40.7% | |
| Liverpool John Moores University (LJMU) | 59 | 52.2% | |
| University of Central Lancashire (UCLAN) | 8 | 7.1% | |
|  |  |  | |
| **Responses to PILL-5** |  |  | |
| ***Pills stick in my throat*** | **113** | **100.0%** | |
| Never | 21 | 18.6% | |
| Almost Never | 44 | 38.9% | |
| Sometimes | 44 | 38.9% | |
| Almost Always | 2 | 1.8% | |
| Always | 2 | 1.8% | |
| ***Pills stick in my chest*** | **113** | **100.0%** | |
| Never | 62 | 54.9% | |
| Almost Never | 34 | 30.1% | |
| Sometimes | 16 | 14.2% | |
| Almost Always | 1 | 0.9% | |
| Always | 0 | 0.0% | |
| ***I have fear of swallowing pills*** | **113** | **100.0%** | |
| Never | 62 | 54.9% | |
| Almost Never | 27 | 23.9% | |
| Sometimes | 20 | 17.7% | |
| Almost Always | 1 | 0.9% | |
| Always | 3 | 2.7% | |
| ***My problem swallowing pills interferes with my ability to take my medicines*** | **113** | **100.0%** | |
| Never | 84 | 74.3% | |
| Almost Never | 17 | 15.0% | |
| Sometimes | 10 | 8.8% | |
| Almost Always | 0 | 0.0% | |
| Always | 2 | 1.8% | |
| ***I can’t take my pills without crushing, coating, or using other forms of assistance*** | **113** | **100.0%** | |
| Never | 91 | 80.5% | |
| Almost Never | 10 | 8.8% | |
| Sometimes | 6 | 5.3% | |
| Almost Always | 0 | 0.0% | |
| Always | 6 | 5.3% | |
|  |  |  | |
| **Responses to 'What materials are already being taught in your current degree'** | **113** | **100.0%** | |
|  |  |  | |
| How to discuss with patients about medication associated side effects | 112 | 99.1% | |
| How to teach patients about effective inhaler use techniques | 111 | 98.2% | |
| How to discuss with patients about medication adherence and missed doses | 109 | 96.5% | |
| How to teach patients about safe medication storage | 98 | 86.7% | |
| How to discuss with patients about medication modification including splitting solid oral dosage forms | 61 | 54.0% | |
| How to determine if a patient has a physiological related/disease related swallowing difficulty | 53 | 46.9% | |
| How to determine if a patient has non-[physiological related/disease related swallowing difficulties | 34 | 30.1% | |
| How to teach patients to swallow tablets, capsules, pulls if they are experiencing difficulty | 23 | 20.4% | |
| How to administer injections to patients | 15 | 13.3% | |
