# Supplementary material for: KidzMed e-learning to upskill student pharmacists to teach pill swallowing to children
Source: PLoS One. 2023 Mar 16;18(3):e0282070. doi: 10.1371/journal.pone.0282070 (PMC10019696; doi:10.1371/journal.pone.0282070)
Supplement: S2 Table — (DOCX) [file pone.0282070.s002.docx]

| Table 2. Summary of data from post-learning questionnaire **Responses** | n | % |
| --- | --- | --- |
| **The learning material means that I will be able to teach the following patients how to swallow pills** | 65 | 100% |
| Adults only | 0 | 0% |
| Children only | 14 | 22% |
| Both adults and children | 51 | 78% |
| **Having completed the virtual learning package I feel comfortable in teaching patients how to swallow pills** | 65 | 100% |
| Strongly agree | 13 | 20% |
| Agree | 49 | 75% |
| Neither agree or disagree | 3 | 5% |
| Disagree | 0 | 0% |
| Strongly disagree | 0 | 0% |
| **Having completed the virtual learning package I feel comfortable in counselling parents or carers about pill swallowing** | 64 | 98% |
| Strongly agree | 12 | 19% |
| Agree | 48 | 75% |
| Neither agree or disagree | 4 | 6% |
| Disagree | 0 | 0% |
| Strongly disagree | 0 | 0% |
| **I feel that completing learning about teaching patients to swallow pills is useful** | 64 | 98% |
| Strongly agree | 46 | 72% |
| Agree | 17 | 27% |
| Neither agree or disagree | 1 | 2% |
| Disagree | 0 | 0% |
| Strongly disagree | 0 | 0% |
| **I would like to put my learning into practise in a classroom setting** | **63** | 97% |
| Strongly agree | 25 | 39% |
| Agree | 30 | 47% |
| Neither agree or disagree | 8 | 13% |
| Disagree | 0 | 0% |
| Strongly disagree | 0 | 0% |
| **Complete this sentence: The pre-session learning was...** | 65 | 100% |
| Too short | 0 | 0% |
| Just right | 64 | 100% |
| Too long | 1 | 2% |
| **Complete this sentence: The pre-session learning was...** | 65 | 100% |
| Too basic | 4 | 6% |
| About right | 61 | 95% |
| Too advanced | 0 | 0% |
| **How satisfied were you with the event?** | 65 | 100% |
| Extremely satisfied | 14 | 22% |
| Very Satisfied | 44 | 69% |
| Somewhat satisfied | 7 | 11% |
| Not so satisfied | 0 | 0% |
| Not at all satisfied | 0 | 0% |
| **Likelihood of recommending this to a friend** | 65 | 100% |
| Promotors | 30 | 46% |
| Passives | 28 | 43% |
| Distractors | 7 | 11% |
