# Supplementary material for: KidzMed e-learning to upskill student pharmacists to teach pill swallowing to children
Source: PLoS One. 2023 Mar 16;18(3):e0282070. doi: 10.1371/journal.pone.0282070 (PMC10019696; doi:10.1371/journal.pone.0282070)
Supplement: S3 Table — (DOCX) [file pone.0282070.s003.docx]

### Table 3. Free-text responses from learners after completing the KidzMed e-learning

| **What did you like most about the intervention** | **What did you like least about the intervention** |
| --- | --- |
| Useful skill to learn | Nothing |
| The demonstration on how to conduct a tablet swallowing consultation | Could have a printable summary sheet to take away. |
| the content was clear and the video showed a clear example of how to teach swallowing tablets | Nothing |
| Learning a new skill that we would otherwise missed out on. | nothing |
| Taught something new | colour scheme, bit too strong on the eyes! |
| interactive learning | Unable to practice yourself |
| The video showing how to talk the children through taking the tablets and how to encourage them even if they are unable to swallow larger tablets. | occasionally repeated itself |
| Interactive | nothing |
| Easy and simple to follow | Nothing |
| quick and easy to access learning resource | nothing |
| The different formats the information was available in | Nothing |
| Easy to follow, short | Nothing |
| teaching child how to take pills | Not as engaging as it would be in person |
| Nice webpage design | Some of the images in the quiz weren't too clear, could be bigger. |
| Easier to engage, good visuals | was not an option button to take you back to the main page to do the next part of the exercise |
| very easy to follow and is aimed at all levels of HCP | There wasn't a lot of solutions about what to do if medications can not be taken. i.e What is the patient has Dysphagia a source on were to find examples of medication you can or cant crush would be helpful. |
| The easy to use website. | not sure |
| Demonstration video | nothing |
| the video demonstration | The quiz's asked for multiple answers and only allowed you to choose 1(e.g. Q7 bottles) and some questions were repeated. |
| Video demonstration | nothing |
| the active online learning session | the functionality of the e-learning page at times (when i clicked links to external pages e.g. the certificate, i would lose all progress on the e-land have to do it again) |
| Easy to follow learning, visual aids, simple language that could be used within the training session with patients | hard to follow the stages of the process |
| Short but useful | The platform was a little difficult to navigate, would be better if it just flowed without having to go back to the start page |
| short and easy to follow with key points covered | A little hard to navigate through with all the different windows and the ‘Next' button didn't show up for me after the test. |
| The videos showing an example of how to conduct the session. | Having to read the introduction. This could have been a bit more brief. |
| Format of e-learning it was engaging and about the right length |  |
| How interactive it was. |  |
| Concise teaching |  |
| you can see correct answers when testing your learning |  |
| quick and to the point |  |
| Easy to do in a short time |  |
| I liked the video that we were given to watch as I thought that the demonstration was useful |  |
| interactive and quick way to learn something new |  |
| It was very shocking to see that some oral medication can be almost 30x the price of a tablet. |  |
| Videos were easy to watch and were informative |  |
| quick and to the point |  |
| The official resource was well formatted and so easy to follow |  |
| very interesting |  |
| how simple is the programme |  |
| opportunity to gain new knowledge I can use in practice |  |
| quick and easy |  |
| short and interactive |  |
| videos and immediate testing of knowledge |  |
| The online e-learning tool was great |  |
| Short so keeps your attention span. |  |
| The e-learning with the videos. |  |
